# Ectopic Expression of BraYAB1-702, a Member of YABBY Gene Family in Chinese Cabbage, Causes Leaf Curling, Inhibition of Development of Shoot Apical Meristem and Flowering Stage Delaying in *Arabidopsis thaliana*

**DOI:** 10.3390/ijms140714872

**Published:** 2013-07-16

**Authors:** Xin-Ling Zhang, Ze-Ping Yang, Jing Zhang, Lu-Gang Zhang

**Affiliations:** 1 College of Horticulture, Northwest A&F University, Yangling 712100, China; E-Mails: zxl99988@126.com (X.-L.Z.); zpyang0327@163.com (Z.-P.Y.); 111zhangjing@163.com (J.Z.); 2 State Key Laboratory of Crop Stress Biology in Arid Areas, Northwest A&F University, Yangling 712100, China; 3 Key Laboratory of Horticulture Plant Biology and Germplasm Create in Northwest China, Ministry of Agriculture, Yangling 712100, China

**Keywords:** Chinese cabbage (*Brassica campestris* L. ssp. *pekinensis* (Lour.) Olsson), YABBY gene family, quantitative analysis, subcellular localization, ectopic expression

## Abstract

YABBY gene family plays an important role in the polarity development of lateral organs. We isolated the BraYAB1-702 gene, a member of the YABBY gene family, from young leaves of Chinese cabbage line 06J45. The full-length gene has a 937 bp CDNA sequence and contains an open reading frame (ORF) of 702 bp. The subcellular localization analysis showed that the expression product of the gene was localized in the nucleus. Ectopic expression of BraYAB1-702 in *Arabidopsis thaliana* caused leaf curling from the adaxial epidermises to abaxial epidermises; the partial abaxialization of the adaxial epidermises of leaves; leaf trichomes and stomata numbers being significantly increased; the plants being severely stunted; the flowering stage being remarkably delayed and inhibiting the development of shoot apical meristem (SAM) with the down-regulation of the expression of SHOOT MERISTEMLESS (STM), Brevipedicellus (BP) and KNAT2 which were related to the development of shoot apical meristem. These results from the present research help to reveal the molecular mechanism of BraYAB1-702 gene in the establishment of adaxial–abaxial polarity of the lateral organs in Chinese cabbage.

## 1. Introduction

In higher plants, lateral organs are initiated from the peripheral zone of shoot apical meristem (SAM). The adaxial–abaxial polarity is established in the development of lateral organs. The YABBY gene family is involved in adaxial–abaxial polarity [1]. At present, six genes that belong to the YABBY gene family have been isolated from *Arabidopsis thaliana*: FILAMENTOUS FLOWER (FIL), YAB2, YAB3, INNERNOOUTER (INO), YAB5 and CRABSCLAW (CRC) [2–4]. In addition, members of the YABBY gene family have also been isolated from *Antirrhinum majus* [5], *Oryza stative* L. [6], *Zea mays* L. [7] and *Triticum aestivum* L. [8], demonstrating the existence of YABBY genes in dicotyledons and monocotyledons.

YABBY gene family is specific to plants. The YABBY proteins are characterized by two conserved domains, a C_2_C_2_ zinc finger-like domain in the N terminus and a YABBY domain in the *C* terminus. No sequence homology is found outside the zinc finger-like domain and the YABBY domain. Although all of these genes are expressed in the abaxial regions of lateral organs, each has the specific expression pattern [2,3,9]. The genes FIL, YAB2 and YAB3 are expressed in cotyledons, leaves and floral organs [2], whereas the expression regions of CRC and INO are only restricted to carpels and nectarines or outer integuments, respectively [3,10]. The expression of the YABBY gene family in the abaxial regions of lateral organs determines the abaxial destiny of the cells [11], promotes the growth and expansion of lateral organs [3,10], and inhibits the transcription of the maintenance gene of SAM, KNOX1, in the lateral organs [12]. Ectopic expression of *Arabidopsis thaliana* YABBY genes leads to partial abaxialization of adaxial sides of lateral organs [2]. The over expression of FIL and YAB3 results in the abaxialization of leaves [2], indicating that FIL and YAB3 promote the abaxial sides’ characteristics of the leaves. Although the YABBY genes in *Arabidopsis thaliana* are related to abaxial sides’ characteristics, they are expressed in the adaxial sides of the leaves in maize, which is a monocotyledon [13]. OsYAB1 gene in rice is not expressed in a polar manner in lateral organs, although it is the homology to YAB2 gene in *Arabidopsis thaliana* [6]. Taken together, these results indicate that the expression and functions of YABBY genes is not entirely conserved in all species, and have diverged between monocotyledons and dicotyledons [14].

Chinese cabbage (*Brassica campestris* L. ssp. *pekinensis* (Lour.) Olsson) originated from China, as one of the vegetable cultivars and possesses the largest cultivated area among all vegetable crops in China. Leaves are the main product organs of Chinese cabbage. So, it is of high significance to study the role of YABBY gene in polarity establishment of the leaf in Chinese cabbage. In this study, the BraYAB1-702 gene was cloned from the young leaves of Chinese cabbage line 06J45. We obtained the transgenic *Arabidopsis thaliana* with the over-expression of BraYAB1-702 by using the floral dip method. Then quantitative analysis and scanning electron microscope were used on the transgenic *Arabidopsis thaliana*, in order to deepen the understanding of the functions of this gene and explore new materials for the study of the development of Chinese cabbage leaves.

## 2. Results and Discussion

### 2.1. Cloning and Sequence Analysis of BraYAB1-702 Gene

**Figure 1:**
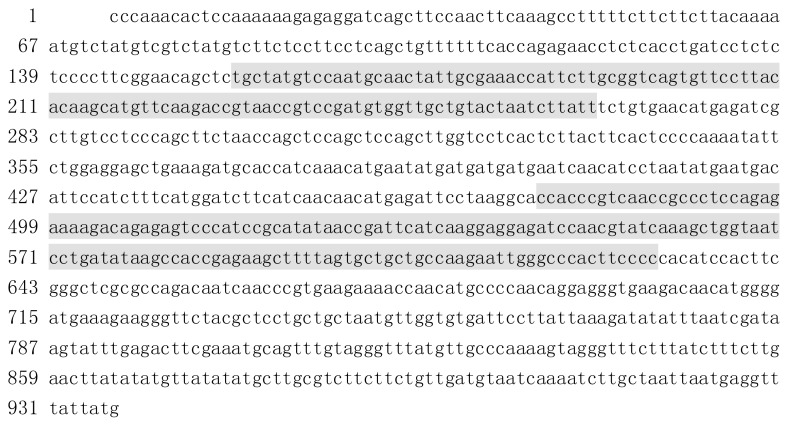
Gene sequence of BraYAB1-702 from Chinese cabbage.

Using homologous cloning, the gene sequence of 937 bp in length was obtained. The open reading frame (ORF) was 702 bp in length (capital letters in the Figure 1). The shaded area at 157–264 bp in the upstream of the ORF contained a C_2_C_2_ zinc finger-like domain; the shaded area at 475–630 bp at the downstream of the ORF was where a YABBY domain was located (Figure 1). The gene was named BraYAB1-702, with GenBank Accession No. JQ828987. The comparison on the National Center for Biotechnology Information (NCBI) website showed a 99% similarity with the DBC43-3-2 gene of *Brassica campestris* L. ssp. *pekinensis* (Lour.) Olsson, and an 89% similarity with *Arabidopsis thaliana* axial regulator YABBY1 (FIL).

**Figure 2:**
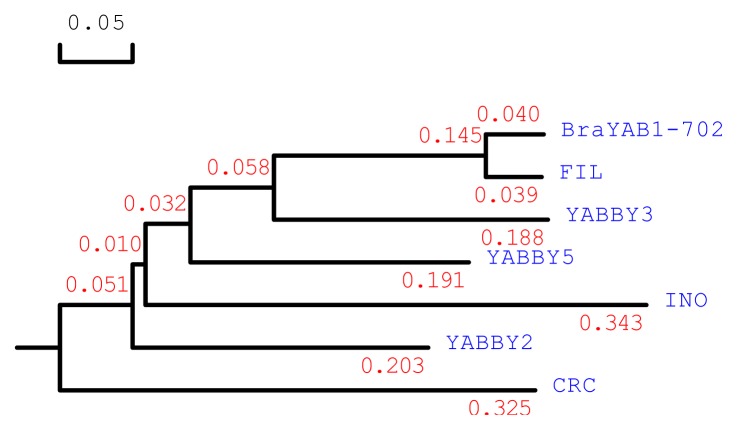
Phylogenetic tree analysis of BraYAB1-702. GenBank accession number of the involved genes for phylogenetic tree analysis are as follows: BraYAB1-702 (JQ828987), FIL (AAD33715), YABBY2 (AAD33716), YABBY3 (AAD33717), INO (AAF23754), YABBY5 (NM179750), CRC (AAD30526). The length of the branch line indicates the extent of difference according to the scale at upper left.

Based on amino acid sequence alignment, a phylogenetic tree was constructed. The dendrogram shows that BraYAB1-702 is more closely related to FIL in *Arabidopsis thaliana*, indicating that BraYAB1-702 is a putative member of the YABBY gene family (Figure 2).

### 2.2. Analysis of Promoter Structure of BraYAB1-702 Gene from Chinese Cabbage and FIL Gene from *Arabidopsis thaliana*

**Figure 3:**
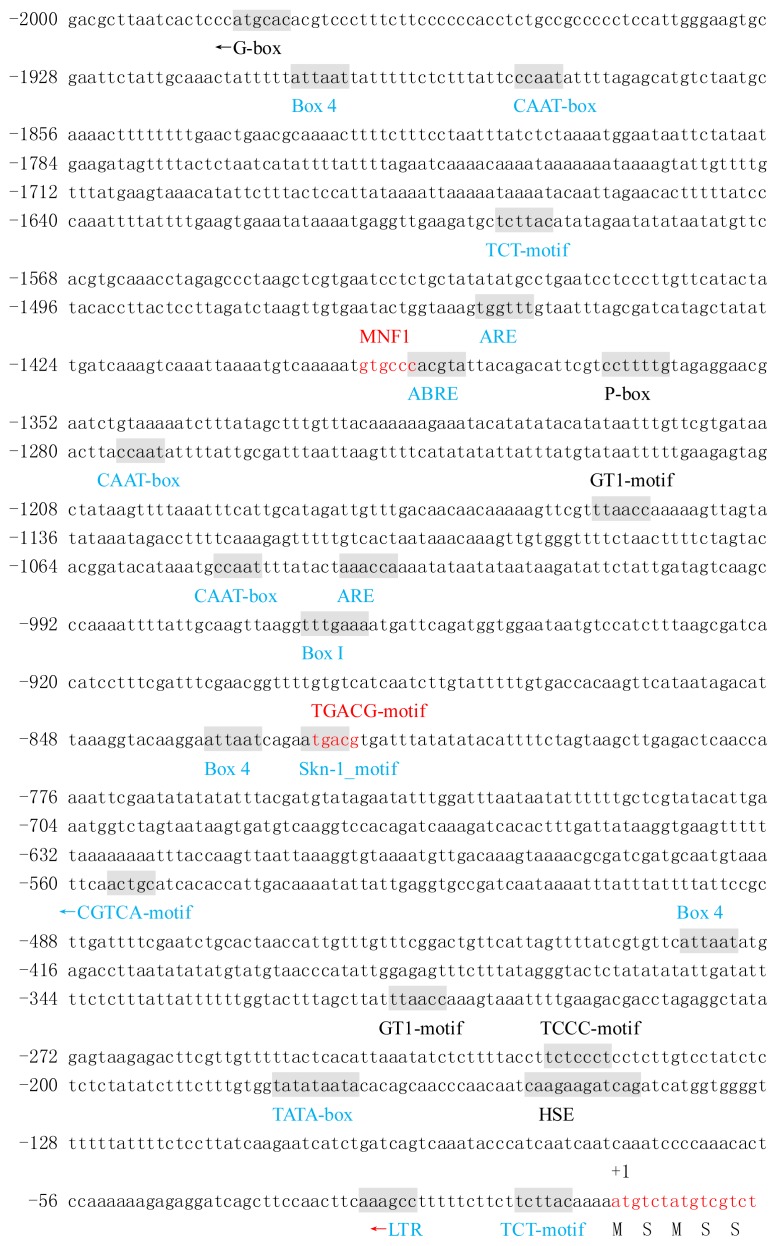
The upstream nucleotide sequence of BraYAB1-702 gene from Chinese cabbage and the predicted cis-acting regulatory elements. The partial sequence of transcription initiation under the (+1) are shown in red letters. Motifs with significant similarity to the previously identified cis-acting elements are shaded and the names are given under the elements or on the elements. The same cis-acting elements between Chinese cabbage BraYAB1-702 gene and *Arabidopsis* FIL gene promoter sequence are marked in blue, the unique cis-acting elements of their own are marked in black and the cis-acting elements overlapping compared to another cis-acting element are marked in red. The direction of the arrowhead represents the direction of action of the cis-element and the direction of action of cis-elements without being marked by arrowhead is the same as the direction of the promoter.

**Figure 4:**
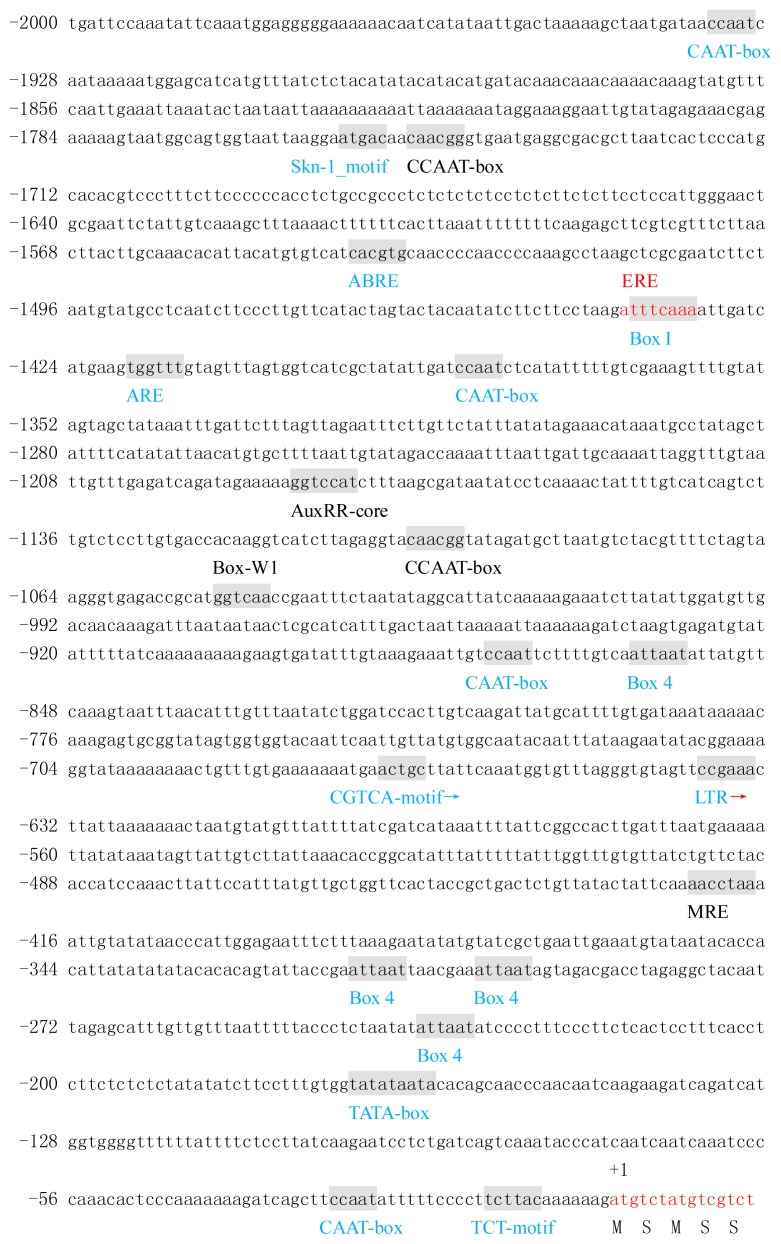
The upstream nucleotide sequence of FIL gene from *Arabidopsis thaliana* and the predicted cis-acting regulatory elements. The partial sequence of transcription initiation under the (+1) is shown in red letters. Motifs with significant similarity to the previously identified cis-acting elements are shaded and the names are given under the elements or on the elements. The same cis-acting elements between *Arabidopsis* FIL gene and Chinese cabbage BraYAB1-702 gene promoter sequence are marked in blue, the unique cis-acting elements of their own are marked in black and the cis-acting element overlapping compared to another cis-acting element is marked in red. The direction of the arrowhead represents the direction of action of the cis-element, and the direction of action of cis-elements without being marked by arrowhead is the same as the direction of the promoter.

The promoter sequence of BraYAB1-702 gene shared a 55.35% identity with the promoter sequence of FIL gene (Figures 3 and 4). The two gene promoters have a large number of cis-acting regulatory elements closely related to light, temperature and hormone responsiveness, besides common TATA-box and CAAT-box. However, there are some differences between the two promoters.

In the light response, two promoters all contain the cis-acting elements Box I, Box 4 and TCT-motif, but the number of these cis elements is different. The promoter of BraYAB1-702 gene has three Box4s and two TCT-motifs, but the promoter of FIL gene has four Box4s and one TCT-motif. In addition, the promoter of the BraYAB1-702 gene also has its own unique cis-acting elements—two GT1-motifs, one MNF1, one TCCC-motif and one G-box—that its action direction is opposite compared with its promoter sequence. The promoter of FIL gene has its own unique cis-acting element MRE.

In the temperature response, two promoters both have low temperature responsive (LTR) elements, but the two cis elements act in the opposite direction, respectively. In addition, the promoter of BraYAB1-702 alone has HSE, a heat shock responsive element.

In response to hormones, the two promoters both have the cis-acting element ABRE related to the abscisic acid responsiveness and the CGTCA-motif, a cis-acting element involved in the MeJA-responsiveness. In addition, the promoter of the BraYAB1-702 gene also has its own unique cis-acting element P-box that associated with gibberellin responsiveness and TGACG-motif, another cis-acting element involved in the MeJA-responsiveness. The promoter of FIL gene has its own unique cis-acting element AuxRR-core involved in the auxin responsiveness, while the cis-acting element ERE related to ethylene responsiveness.

The two promoters both have ARE, a cis-regulatory element essential for the anaerobic induction, and Skn-1_motif, a cis-regulatory element for the endosperm expression. In addition, the promoter of FIL gene from *Arabidpsis* alone has Box-W1, a cis-regulatory element related to fungal elicitor responsiveness, and CCAAT-box, a cis-acting element combined with the transcription factor MYBHv1.

In short, there is not only the same but also the different cis elements between promoters of BraYAB1-702 gene from Chinese cabbage and FIL gene from *Arabidopsis*; even with the same cis-acting element, the number of them is different and the direction may be opposite. These results suggest that the regulation of expression of these two genes have a certain conservation and the respective specificity.

### 2.3. Subcellular Localization Analysis of BraYAB1-702

**Figure 5:**
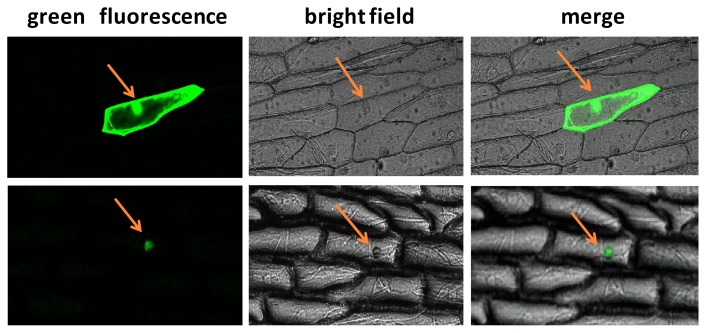
Localization of the product expressed by the BraYAB1-702 gene of Chinese cabbage in the nucleus of the onion epidermal cell (Scale bar: 100 μm). Upper panels: in the negative control group, the epidermal cell of onion transformed with GFP2 vector (35S::GFP); Lower panels: the epidermal cell of onion transformed with 35S::BraYAB1-702-GFP fusion vector.

**Figure 6:**
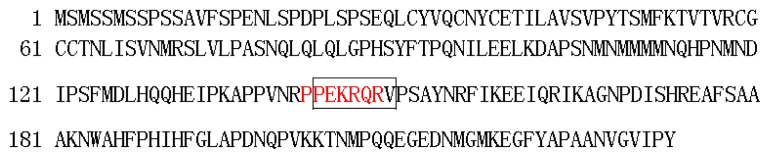
Analysis of the nuclear localization signal of BraYAB1-702 protein sequence.

The results of the subcellular localization analysis showed that the green fluorescence was only observed in the nuclei of the onion epidermal cells transformed by the fusion expression vector 35S::BraYAB1-702-GFP. In the negative control group, the whole epidermal cells of onion transformed by 35S::GFP2 showed green fluorescence (Figure 5). This experiment showed that the product expressed by BraYAB1-702 gene was a transcription factor located in the nucleus. Analysis of subcellular localization showed that the gene was located in the nucleus, which was consistent with the localization result of FIL genes of *Arabidopsis thaliana* [9] and OsYAB1 gene of *Oryza stative* L. [6]. Analysis of the nuclear localization signal in BraYAB1-702 protein sequence (NLS) found that there is a putative nuclear localization signal sequence PPEKRQR at the region from amino acid 142 to 148 (red letters in Figure 6), or a putative nuclear localization signal sequence PEKRQRV at the region from amino acid 143 to 149 (letters in box in Figure 6).

### 2.4. Phenotype Analysis of *Arabidopsis thaliana* Transformed with BraYAB1-702 Gene of Chinese Cabbage

**Figure 7:**
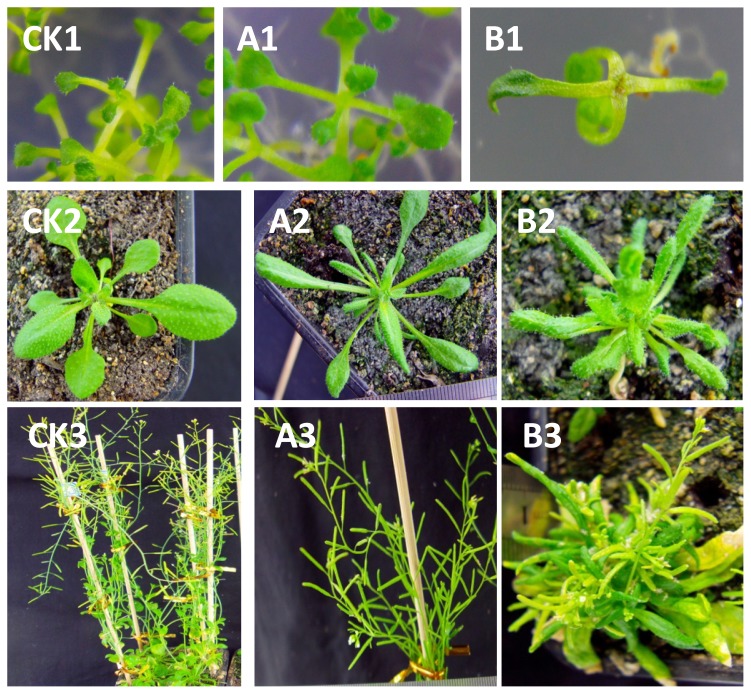
Phenotypic analysis of 35S::BraYAB1-702 transgenic *Arabidopsis thaliana* plants. **CK**: Wild-type *Arabidopsis thaliana*; (**A**) Transgenic plants with mild phenotype; (**B**) Transgenic plants with strong phenotype. 1. Seedling stage; 2. Rosette stage; 3. Flowering and fruiting stage.

**Figure 8:**
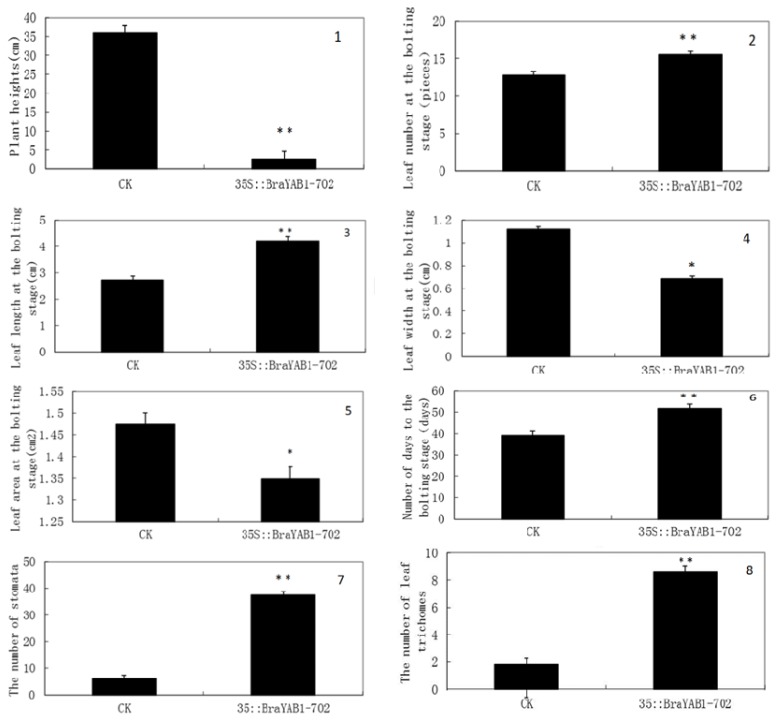
Variance analyses for phenotype of wild type and transgenic *Arabidopsis*. **1**: Plant heights; **2**: Leaf number of the rosette leaf at the bolting stage; **3**: Leaf length of the rosette leaf at the bolting stage; **4**: Leaf width of the rosette leaf at the bolting stage; **5**: Leaf area of the rosette leaf at the bolting stage; **6**: The number of days to the bolting stage; **7**: The number of stomata; **8**: The number of leaf trichomes; CK: Wild type; 35S::BraYAB1-702: Transgenic *Arabidopsis* with BraYAB1-702 gene; * The difference was significant as compared to wild type (*p* < 0.05); ** The difference was extremely significant as compared to wild type (*p* < 0.01).

There are three classes of phenotypes for *Arabidopsis thaliana* transformed with the BraYAB1-702 gene. Class I, which had a mild phenotype, showed that the leaves were mildly curled and became long-narrow; SAM was slightly inhibited; the plants were capable of bolting, flowering and bearing fruit; the height of the bolt was the same as that of the wild type and the flowering stage was clearly delayed (Figure 7A). Class II, which had a strong phenotype, showed that the leaves were more severely curled; SAM was strongly inhibited (Figure 7B1): the plants were severely stunted; the flowering stage was more clearly delayed; the rosette leaves gathered closely at the base during the bolting stage; the flower stalk was short and grew closer to the base; the boat was unable to elongate normally; the leaf distance and pod distance were short; the plants were only 2–4 centimeters high during the fruiting stage. The difference of plant heights was extremely significant as compared to wild type (*p* < 0.01) (Figure 8-1). However, the plants with strong phenotype were able to bloom and bear fruit (Figure 7B3). Class III, which had the strongest phenotype, showed that SAM was seriously inhibited: the seedlings have few leaves, even only cotyledons. Although those seedlings with the strongest phenotype were able to survive in MS/2 culture medium, they died soon after being transplanted to the substrate.

**Figure 9:**
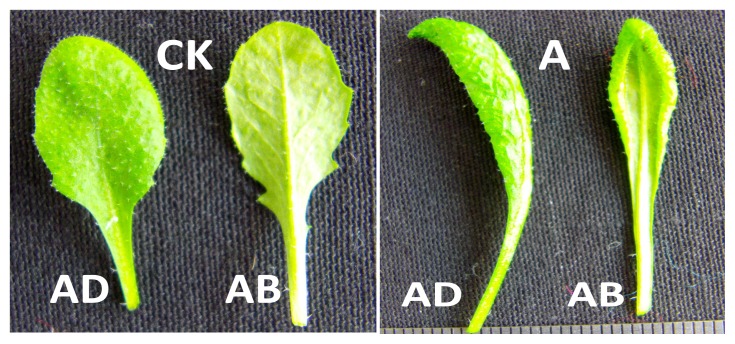
Comparison of the adaxial and abaxial sides of the leaves of wild type and 35S::BraYAB1-702 transgenic *Arabidopsis thaliana*. **CK**: Leaves of wild type; (**A**) Leaves of transgenic *Arabidopsis thaliana*; (**AD**) Adaxial side of leaf; (**AB**) Abaxial side of leaf.

Figure 9 shows that the leaves of wild-type *Arabidopsis thaliana* are flat and exhibit oval in shape. The rosette leaves of BraYAB1-702 gene-modified *Arabidopsis thaliana* are long-narrow and have a pronounced epinastic (downward curling). That is to say, the rosette leaves of transgenic plants curl towards the abaxial side dramatically in contrast with those of wild-type plants.

At the bolting stage, the average value of leaf number, leaf length, leaf width, leaf area in wild-type *Arabidopsis* were 12.8 ± 0.50 pieces, 2.72 ± 0.16 cm, 1.12 ± 0.02 cm, 1.47 ± 0.03 cm^2^; while the average value of leaf number, leaf length, leaf width, leaf area in transgenic *Arabidopsis* were 15.6 ± 1.29 pieces, 4.22 ± 0.10 cm, 0.69 ± 0.13 cm, 1.35 ± 0.003 cm^2^. As we can see from the above data, at the bolting stage, the average leaf number of the transgenic *Arabidopsis* is about 2.8 pieces more than that of wild type and the difference is extremely significant between them (*p* < 0.01) (Figure 8-2), leaf length is about 1.6 times than that of wild type with extremely significant difference (*p* < 0.01) (Figure 8-3), leaf width is about 0.6 times than that of wild type with significant difference (*p* < 0.05) (Figure 8-4), leaf area is approximately 0.9 times than that of wild type with significant difference (*p* < 0.05) (Figure 8-5). It can be concluded that, compared with the wild-type *Arabidopsis*, the leaves of transgenic *Arabidopsis* are more long-narrow and the growth potential is much weaker.

**Figure 10:**
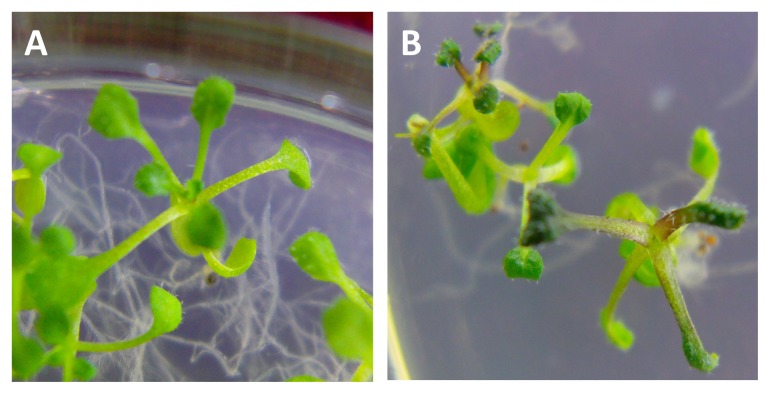
The purple seedlings of BraYAB1-702 gene-modified *Arabidopsis thaliana*. (**A**) Wild-type *Arabidopsis thaliana*; (**B**) Transgenic *Arabidopsis thaliana*.

Interestingly, some purple transgenic seedlings were found in the transgenic *Arabidopsis thaliana* with ectopic expression of BraYAB1-702 of Chinese cabbage, which with purple petiole and dark green leaves (Figure 10). However, the proportion of purple seedlings is small, and with the growth of seedlings, the purple gradually fades. In the late growth stage, people can be seen only in the leaf petiole. The FIL gene in *Arabidopsis thaliana* is the homologous gene of the BraYAB1-702 in Chinese cabbage. Bonaccorso *et al*. [15] and Siegfried *et al*. [2] also found a similar phenomenon in FIL over expressed *Arabidopsis thaliana*, they presume it may be caused by the accumulation of anthocyanins; Zhao *et al*. [8] also found purple seedlings in severe phenotype *Arabidopsis thaliana* with ectopic expression of wheat TaYAB1 genes. However, it is unclear how the over expression of BraYAB1-702 triggers the synthesis and accumulation process of anthocyanin.

**Figure 11:**
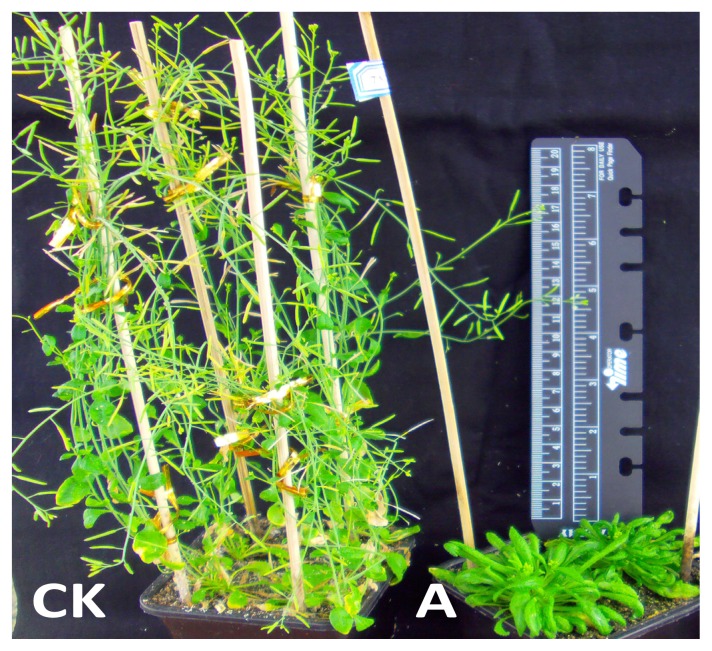
Comparison of wild type (**CK**) and transgenic (**A**) *Arabidopsis thaliana* during flowering stage.

Figure 11 shows that the vegetative stage of BraYAB1-702 gene-modified *Arabidopsis thaliana* was clearly prolonged, leaf senescence was inhibited and its flowering stage was delayed (Figure 11A). When the flowering stage of wild-type *Arabidopsis thaliana* (Figure 11CK) came to an end, the transgenic *Arabidopsis thaliana* was still in the end period of rosette stage and just began to bolt. Under a 14 h light/10 h dark cycle, the flowering stage of BraYAB1-702 gene-modified *Arabidopsis thaliana* was delayed by about 5–20 days compared to the wild type. The difference of the number of days to the bolting stage was extremely significant as compared to wild type (*p* < 0.01) (Figure 8-6).

### 2.5. Ectopic Expression of BraYAB1-702 in *Arabidopsis thaliana* Promotes Abaxialization of Adaxial Epidermises in Leaves

**Figure 12:**
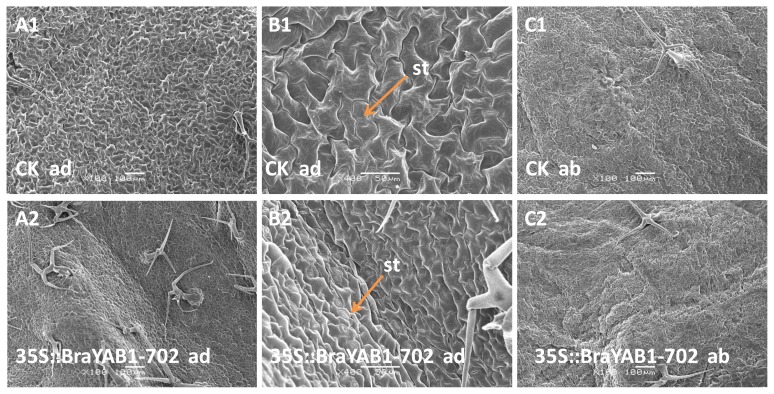
Scanning electron microscopic analysis of leaves of 35S::BraYAB1-702 transgenic *Arabidopsis thaliana*. (**A1**) Adaxial epidermis of a wild-type rosette leaf (×100); (**B1**) Adaxial epidermis of a wild-type rosette leaf (×400); (**C1**) Abaxial epidermis of a wild-type rosette leaf (×100); (**A2**) Adaxial epidermis of a transgenic rosette leaf (×100); (**B2**) Adaxial epidermis of a transgenic rosette leaf (×400); (**C2**) Abaxial epidermis of a transgenic rosette leaf (×100); ad: Adaxial epidermis of a rosette leaf; ab: Abaxial epidermis of a rosette leaf; St: Stomata. Bars in (**A1**), (**C1**), (**A2**) and (**C2**) are 100 μm and bars in (**B1**) and (**B2**) are 50 μm.

To determine whether the ectopic expression of BraYAB1-702 affects the adaxial–abaxial polarity of leaves, the epidermises of transgenic rosette leaves were examined under scanning electron microscope. The adaxial epidermises of wild-type leaves have a flat surface (Figure 12A1), while the wild-type abaxial epidermises have an undulating surface (Figure 12A2). In addition, a high density of stomata was observed on the adaxial epidermises in transgenic leaves (Figure 12B2) and the number of stomata in transgenic leaves was increased by about 6–9 times than that of the wild type. The difference of stomata numbers was extremely significant as compared to wild type (*p* < 0.01) (Figure 8-7). Furthermore, the number of leaf trichomes in the adaxial epidermises of transgenic *Arabidopsis* significantly increased (Figure 12A2) compared with the wild-type ones (Figure 12A1). The difference of leaf trichomes numbers was extremely significant as compared to wild type (*p* < 0.01) (Figure 8-8). The result indicates that at the adaxial epidermis of rosette leaves of 35S::BraYAB1-702 transgenic *Arabidopsis thaliana* (Figure 12A2), present characteristics of the epidermal cells partly similar to those of the abaxial epidermis of rosette leaves of wild-type ones (Figure 12C1).

In this study, we transferred the BraYAB1-702 gene of Chinese cabbage to *Arabidopsis* plants. The ectopic expression of BraYAB1-702 in *Arabidopsis* promotes the abaxialization of the adaxial epidermises in leaves and arrests the SAM development. The similar phenotypes were previously reported in transgenic *Arabidopsis* plants with ectopic expression of either FIL or YAB3 [2]. Because BraYAB1-702 is more closed to FIL in *Arabidopsis* by phylogenetic analysis, it is most likely that BraYAB1-702 resembles FIL, which play an important role in determining the abaxial cell fate in the regulatory pathway of adaxial–abaxial polarity when ectopically expressed in *Arabidopsis*. The results suggest that BraYAB1-702 in Chinese cabbage has the similar function of the YABBY genes in *Arabidopsis*.

### 2.6. Real-Time Quantitative PCR of BraYAB1-702 Transgenic *Arabidopsis thaliana*

**Figure 13:**
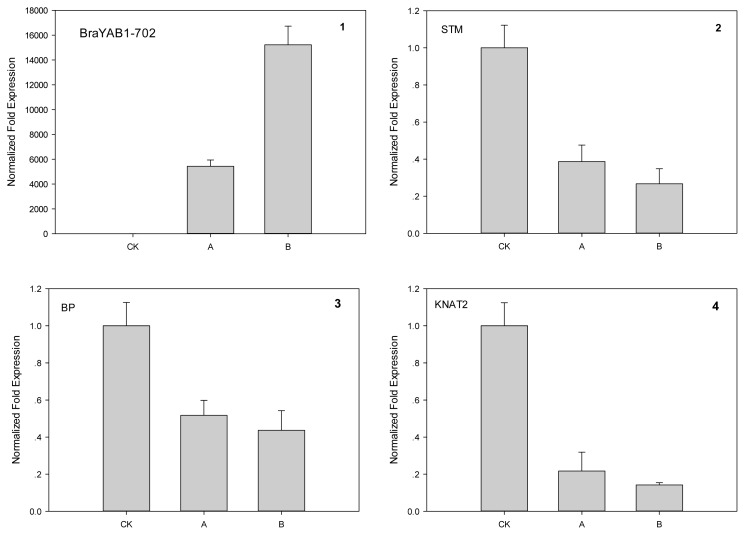
Transcript levels of BraYAB1-702, STM, BP and KNAT2 in wild-type plants and 35S::BraYAB1-702 transgenic plants (**1**–**4**). CK: Wild-type seedlings; A: Transgenic seedlings with slightly curled leaves; B: Transgenic seedlings with strongly curled leaves.

To determine the expression levels of BraYAB1-702, STM, BP and KNAT2 in 35S::BraYAB1-702 transgenic plants, real-time quantitative PCR were performed. The BraYAB1-702 gene is not expressed in wild-type *Arabidopsis* as the control (Figure 13-1). BraYAB1-702 transcripts accumulate in transgenic plants, and the phenotypic severity of transforms is consistent with the expression levels of BraYAB1-702. The expression of the strong phenotype was about 2.8 times than that of the mild phenotype. The larger the expression of BraYAB1-702, the more severely inhibited SAM, the more curled the leaves and the more stunted the plants would be (Figure 7B).

In order to analyze the molecular foundation of the inhibitory effect imposed by the over expression of BraYAB1-702 in SAM of *Arabidopsis thaliana*, real-time quantitative PCR was performed on the STM, BP and KNAT2 genes related to the development and growth of SAM of transgenic *Arabidopsis thaliana*. The results showed that the STM gene expression in the transformant was down-regulated by 2.6–3.7 times as compared to wild type (Figure 13-2A,B), the expression of BP gene was down-regulated by 1.9–2.3 times (Figure 13-3A,B) and the expression of KNAT2 gene was down-regulated by 4.6–7.0 times (Figure 13-4A,B). The quantitative analysis indicated that the over expression of BraYAB1-702 gene inhibited the expression of STM, KNAT2 and BP genes related to SAM. The expression of BraYAB1-702 genes was directly related to the mild and the strong phenotype of transgenic *Arabidopsis thaliana*. When the expression of BraYAB1-702 genes was low, the inhibitory effect on the seedlings of transgenic *Arabidopsis thaliana* was slight, the curling degree of leaves and the dwarfing degree of the plants were low as well. When the expression of BraYAB1-702 gene was high, SAM of the seedlings of transgenic *Arabidopsis thaliana* was severely inhibited, with strongly curled leaves and dwarfed plants.

The KNOX gene family expressed in SAM and was crucial for the maintenance and growth of the latter [16–19]. As a member of the KNOX gene family, STM gene plays an important role in the maintenance of SAM and in the initialing of the lateral organ [17,20,21]. In the leaves of mutants with the absence of FIL and YAB3 activities, the transcription level of KNOX1 gene was up-regulated. Similar to the phenotype with over expression of KNOX1, meristem grew on the edge of the cotyledons and leaves in full yab3 double mutant [12]. This indicated that YABBY gene inhibited the development of SAM. In this study, the results of quantitative analysis showed that the transcription level of STM gene in 35S::BraYAB1-702 transgenic *Arabidopsis thaliana* dropped significantly (Figure 13-2). This was likely caused by the over expression of BraYAB1-702, resulting directly or indirectly in the suppression of STM gene transcription. Thus, the development of SAM was restricted (Figure 7B1).

BP (KNAT1) and KNAT2 genes are also the members of the KNOX gene family [12]. BP gene is related to the morphogenesis of the stem and internodes [22] and its function is partially redundant with STM [23]. KNAT2 gene involved in the development of carpel [24] and has a function of synergism interactively with cytokinin [25]. In this experiment, the over expression of the BraYAB1-702 gene of Chinese cabbage induced the drop of the transcription level of the BP gene in transgenic *Arabidopsis thaliana* (Figure 13-3). This could be one of the reasons for inhibiting growth and development of the steam Internode and flower stalk Internode of transgenic *Arabidopsis thaliana*. Meanwhile, the over expression of the BraYAB1-702 gene of Chinese cabbage also led to the drop of expression of the KNAT2 gene (Figure 13-4). This could weaken the synergistic effect between KNAT2 and cytokinin, and then inhibited the development of SAM. Thus, the elongation of stem Internode and flower stalk Internode in transgenic *Arabidopsis thaliana* was affected, leading to stunted plants (Figure 7B3).

The over expression of the BraYAB1-702 gene in transgenic *Arabidopsis thaliana* resulted in the prolongation of vegetative stage and delay of flowering stage (Figure 11A) as compared to wild type. This result was consistent with the findings by Zhao *et al*., about the genetic transformation of *Arabidopsis thaliana* by wheat TaYAB2 gene, in which the flowering stage of transgenic *Arabidopsis thaliana* was delayed as compared to wild type [14]. It indicated that BraYAB1-702 gene of Chinese cabbage had the same effect as TaYAB2 gene of wheat. In addition, BraYAB1-702 gene possessed the characteristics of delaying the flowering stage and prolonging the vegetative period, which could be utilized in the vegetable breeding program on the resistance to bolting of spring Chinese cabbage and prolonging the harvest period of vegetative organs, and then achieves the goal of long-term supplying market.

## 3. Experimental Section

### 3.1. Experimental Materials and Cultivation Management

Chinese cabbage line 06J45 was cultivated and provided by Chinese cabbage Study Team at College of Horticulture, Northwest A&F University. The seedlings were sowed at tissue culture room to provide materials for the cloning of the BraYAB1-702 gene. *Arabidopsis thaliana* (Col) was purchased from the *Arabidopsis thaliana* Resource Center of Ohio State University. The seedlings were sowed in the tissue culture room for genetic transformation during bolting period. The temperature of the tissue culture room was kept at 22–25 °C. The 12 h light/12 h dark cycle was used for rosette stage and then switched to a 14 h light/10 h dark cycle after bolting.

### 3.2. Cloning and Sequence Analysis of BraYAB1-702 Gene

The RNA prep pure plant kit produced by Beijing Tianjin Corporation was used to extract the total RNA from young leaves of Chinese cabbage line 06J45. The first strand CDNA synthesis was conducted using PrimeScript 1st strand CDNA synthesis kit (TaKaRa). The homologous cloning primers were designed with Chinese cabbage DBC43-3-2 gene sequence (Accession No. FJ868216) by the template as follows:

Forward primer: 5′ CCCAAACACTCCAAAAAAGAGAGGA 3′,
Reverse primer: 5′ CCCAAACACTCCAAAAAAGAGAGGA 3′.

The PCR amplification conditions were as follows: predegeneration at 94 °C for 3 min, degeneration at 94 °C for 40 s, annealing at 53 °C for 40 s, extension at 72 °C for 70 s, 35 cycles and final extension at 72 °C for 10 min. After amplification, the collected target sequence was connected with pMD18-T vector (TaKaRa, Dalian, China) and submitted for sequencing. After gene cloning, DNAstar software was used for ORF analysis. The homology analysis of nucleotide sequence was performed by blasting search on the NCBI website [26]. Conserved Domain Search Service of NCBI was used for the analysis of functional domains [27]. Combining with BioEdit software (Version 7.0.5; Isis Pharmaceuticals: Carlsbad, CA, USA), these functional domains were located on the corresponding nucleotide sequence. The phylogenic tree was generated using DNAMAN 4.0 software (Lynnon BioSoft Company: Vaudreuil, QC, Canada).

### 3.3. Identification and Structure Analysis of Promoter Sequences

BraYAB1-702 gene was positioned in the Chinese cabbage A04 chromosome of 18606921-18609195 bp in Brassica database [28]. FIL gene was positioned in the chromosome 2 of 18628450-18630552 bp in the *Arabidopsis thaliana* genome database of NCBI [29]. To analysis promoter structure of BraYAB1-702 and FIL, 2000 bp sequence of the 5′ regulatory region before the transcription initiation site was used respectively. The cis-acting elements of promoter sequence were analysed respectively in promoter prediction website PlantCARE [30].

### 3.4. Subcellular Localization

The subcellular localization vector GFP2 was provided by Dr. Wei-Li Guo. Subcellular localization primers of the BraYAB1-702 gene were designed as follows:

Forward primer: 5′ GGCTCTAGAATGTCTATGTCGTCTATG 3′,
Reverse primer: 5′ GCCGGTACCTTAATAAGGAATCACACC 3′.

The 35S::BraYAB1-702-GFP2 fusion expression vector was constructed. The epidermal cells of onion were subjected to ballistic bombardment. After dark culture for 24 h in MS culture medium, confocal laser scanning microscopy was used for photography, and the photos were analysed by NIS Viewer 4.00 b770 (Nikon, Japan). Ballistic PDS-1000/He particle delivery system (Bio-rad, Hercules, CA, USA) and Confocal microscope A1R (Nikon, Japan) were used respectively. Reference Rey *et al*.’s method [31], we have analysed the nuclear localization signal (NLS) on the BraYAB1-702 protein sequence by using the software WoLFPSORT (Human Genome Center, Institute for Medical Science, University of Tokyo, Tokyo, Japan).

### 3.5. Construction and Transformation of the BraYAB1-702 Gene Expression Vector and Screening with Hygromycin

Plant expression vector pWR306 was used (provided by Professor Yue-Jin Wang) to construct pWR306-BraYAB1-702 plant expression vector. Agrobacterium EHA105 was transformed and floral dip method [32] was used to transform *Arabidopsis thaliana* (Col). The seeds of T1 generation were collected and screened on the hygromycin MS/2 culture medium. Resistant plants were subject to inbreeding and the seeds were reserved. The seeds of transgenic *Arabidopsis thaliana* were screened on the hygromycin MS/2 culture medium. The seedlings transformed successfully maintained their vitality on the antibiotic culture medium; the seedlings transformed unsuccessfully first turned yellow and then brown, and finally died. The screening was carried out until T3 generation using hygromycin. Most seeds harvested from individual plants were homozygous. These homozygous seedlings were used for subsequent identification and functional analysis. Leaf number, leaf length, leaf width and leaf area of wild type and transgenic *Arabidopsis thaliana* were measured by using ImageJ1.42 software (National Institutes of Health, Bethesda, MD, USA).

### 3.6. DNA Extraction and DNA-PCR Identification

In order to determine whether the resistant seedlings screened by hygromycin were successfully transformed or not, the genomic DNA of T3 generation of *Arabidopsis thaliana* was extracted for analysis by PCR using specific primers of BraYAB1-702.

DNAsecure plant kit produced by Beijing Tianjin Corporation was used to extract the genomic DNA of wild type and transgenic *Arabidopsis thaliana* plants. Specific primers of the BraYAB1-702 gene were used to analyse the transforms as follows:

Forward primer: 5′ ATGTCTATGTCGTCTATGTCTTCTCC 3′,
Reverse primer: 5′ TTAATAAGGAATCACACCAACATTAG 3′.

The results of gel electrophoresis showed that wild type *Arabidopsis thaliana* of the control group had no target band of BraYAB1-702 genes, while the target band of this gene was detected in the transgenic *Arabidopsis thaliana*. This indicated the success of genetic transformation of *Arabidopsis thaliana* by the BraYAB1-702 gene from Chinese cabbage.

### 3.7. Analysis of BraYAB1-702, STM, BP and KNAT2 Expression by Real-Time PCR

The method described in Section 4.2 was employed to extract total RNA and first strand CDNA synthesis for wild type and transgenic *Arabidopsis thaliana* plants. The primers of BraYAB1-702 and reference gene UBC (Ubiquitin conjugating enzyme) were designed to conduct quantitative analysis of transgenic *Arabidopsis thaliana* plants. To study the molecular mechanism of inhibition of the growth of SAM in transgenic *Arabidopsis thaliana* plants, the primers for STM, BP and KNAT2 genes related to the development of SAM of *Arabidopsis thaliana* and ACTIN8 was used as the reference gene (Table 1) were designed for quantitative analysis. The primers of STM, BP, KNAT2 and ACTIN8 were designed following the method described by Kumaran *et al*. [12]. The data obtained by real-time PCR were processed using SigmaPlot10.0 (Systat Software Inc., San Jose, CA, USA).

### 3.8. Scanning Electron Microscope and Sample Preparation

The samples were prepared according to the Guidelines for Electron Microscope Sample Preparation formulated by State Key Laboratory of Crop Stress Biology in Arid Areas of Northwest A&F University. The model of scanning electron microscope was JSM-6360LV (Japan Electronics). The photos were processed using Adobe Photoshop CS. At the same time, the method reported by Zhao *et al*. [8] was also referred to observe the epidermises of the rosette leaves of 35S::BraYAB1-702 gene-modified *Arabidopsis thaliana* and wild-type plants.

### 3.9. Variance Analyses for Phenotype of Wild Type and Transgenic *Arabidopsis*

In the bolting stage of wild type and transgenic *Arabidopsis*, the number of days to the bolting stage and the number of leaves were recorded, the leaf length, leaf width and leaf area were measured by using ImageJ software. The number of leaf trichomes was counted according to each scanning electron microscope picture (×100) of the rosette leaf epidermis, the number of stomata was counted according to each scanning electron microscope picture (×400) of the rosette leaf epidermis and plant height was measured in the fruiting stage of wild type and transgenic *Arabidopsis*. Variance analyses and mapping for phenotype of wild type and transgenic *Arabidopsis* were performed by using the Microsoft Excel.

## 4. Conclusions

Taken together, BraYAB1-702 is a member of the YABBY gene family and it is expressed in the lateral organs of Chinese cabbage. The products of gene expression were located in the nucleus. The expression of this gene resulted in long-narrow leaves and curling from adaxial sides to abaxial sides in transgenic *Arabidopsis thaliana*. The adaxial epidermis became uneven and the characteristics partly similar to those of the abaxial epidermis of rosette leaves of wild-type *Arabidopsis thaliana*. Leaf trichomes and stomata numbers were significantly increased on the adaxial epidermis of the rosette leaves of 35S::BraYAB1-702 gene-modified *Arabidopsis thaliana*. The flowering stage of the transgenic *Arabidopsis thaliana* plants was delayed, the Internode length became shorter and the plants were severely stunted. The mild or strong phenotype of transgenic *Arabidopsis thaliana* directly corresponded to the low and high expression of BraYAB1-702 genes. Quantitative analysis showed that the expressions of STM, KNAT2 and BP genes related to the development of SAM in *Arabidopsis thaliana* were down-regulated and their extent of decrease were in negative correlation with the expression of BraYAB1-702 genes. This indicated that high expression of BraYAB1-702 possibly caused the decline of expression of STM, KNAT2 and BP. As a result, the development of the SAM of the transgenic *Arabidopsis thaliana* plant was inhibited.

## Figures and Tables

**Table 1:** Primers for quantitative analysis.

Primer name	Forward primer(5′→3′)	Reverse primer(5′→3′)
BraYAB1-702	TGATCCTCTCTCCCCTTCGG	CGGACGGTTACGGTCTTGAA
UBC	TAACTGCGACTCAGGGAATCTT	TCATCCTTTCTTAGGCATAGCG
STM	TGTCAGAAGGTTGGAGCACCA	TTTGTTGCTCCGAAGGGTAA
BP	TCATGGAAGCATACTGTGACA	TGACTCAGAAGGATATGGCCA
KNAT2	GATTGCCAAAAGGTGGGAGC	TGTCGCCTTCAGTAGGGTA
ACTIN8	ATGAAGATTAAGGTCGTGGCA	CCGAGTTTGAAGAGGCTAC
